# Correlating the Depth of Sedation Between the Ramsay Sedation Scale and Bispectral Index Using Either Intravenous Midazolam or Intravenous Propofol in Elderly Patients Under Spinal Anaesthesia

**DOI:** 10.7759/cureus.50763

**Published:** 2023-12-19

**Authors:** Abhinav Sachdeva, Sofia Jaswal, Harsimran S Walia, Y K Batra

**Affiliations:** 1 Cardiac Anaesthesia, Alchemist Hospital, Panchkula, IND; 2 Anaesthesia, Critical Care and Pain, Homi Bhabha Cancer Hospital and Research Center, New Chandigarh, IND; 3 Anaesthesia and Pain Management, Max Superspeciality Hospital, Mohali, IND

**Keywords:** spinal, correlation, bispectoral index, ramsay sedation scale, depth of sedation

## Abstract

Background: Supplementation of spinal anaesthesia with sedatives or anxiolytics has emerged as a standard protocol to alleviate patients' anxiety and to produce amnesia during the surgical procedure. Thus, judicious use of sedation can make surgeries under spinal anaesthesia more comfortable and acceptable for the elderly patient, the surgeon, and the anaesthesiologist. However, over-sedation may jeopardise the safety of the patient. Appropriate sedation helps reduce physiological stress, which leads to a better result. Therefore, monitoring the depth of sedation becomes essential. The Ramsay sedation scale (RSS) and bispectral index (BIS) both are used widely to assess the depth of sedation.

Objectives: The primary objective of the study was to assess and correlate the depth of sedation between the BIS and RSS in elderly patients using midazolam and propofol under spinal anaesthesia. The secondary objectives were to observe any difference in the commencement of sedation between the two groups and to observe haemodynamic changes between the two groups.

Methods: A total of 60 elderly patients undergoing urological procedures under spinal anaesthesia were randomly assigned to receive either midazolam (Group A, n=30) or propofol (Group B, n=30) for sedation. In Group A, patients were given an initial bolus of midazolam 0.03 mg/kg and a maintenance incremental bolus of 0.01 mg/kg up to a maximum of 2.5 mg in 10-minute intervals. Group B used propofol with an initial bolus dose of 0.5 mg/kg over two minutes and a maintenance bolus of 10-20 mg as required for the maintenance of sedation depth. Sedation was titrated to achieve a BIS score of 70-80 and an RSS score of 3-4. Heart rate, non-invasive systolic, diastolic, mean arterial blood pressure, oxygen saturation (SPO2), and the correlation coefficient between the BIS and RSS were measured at 0 (baseline), 5, 10, 20, 30, 40, 50, and 60 minutes of interval.

Results: The correlation coefficient between the BIS and RSS scores in Group A at various time intervals indicate a strong correlation coefficient of -0.76 at five minutes, -0.64 at 20 minutes, -0.78 at 30 minutes, -0.56 at 40 minutes, and -0.39 at 50 minutes. In Group B, the correlation coefficient between the BIS and RSS scores at various time intervals indicate a strong correlation coefficient of -0.75 at five minutes, -0.76 at 20 minutes,-0.64 at 30 minutes, -0.89 at 40 minutes, and -0.46 at 50 minutes of interval. We also observed that the BIS drops to a lower level in patients receiving propofol (Group B) with a significant difference depicting early onset of sedation with propofol. In Group B, HR and MAP were significantly less than those of Group A. There was no significant difference in terms of mean age, sex, and body weight in the patients of both groups.

Conclusion: The BIS and RSS scores indicate a strong correlation with a magnitude of 70%-80%, but more in Group B (propofol) than Group A (midazolam). Therefore, the characteristics of each sedative drug can influence the level of sedation during spinal anaesthesia. Clinicians should use a combination of BIS values and other objective sedative methods to determine the degree of sedation, rather than relying exclusively on BIS values.

## Introduction

The use of spinal anaesthesia is often limited by the unwillingness of patients to remain awake during surgery. Intravenous sedative medications are useful for the same as positioning for surgery can be uncomfortable and spontaneous movements by an inadequately sedated patient can cause interference with the surgical procedure. Therefore, supplementation of spinal anaesthesia with sedatives or anxiolytics has emerged as a standard protocol to alleviate patient’s anxiety and to produce amnesia of the surgical procedure. An ideal supplemental sedative should provide effective anxiolytics, an easily controllable level of sedation, a predictable depth of amnesia, a rapid and clear-headed recovery, and minimal peri-operative side effects with no evidence of cumulation. Sedation relieves anxiety and enhances patient comfort when combined with regional anesthesia [1]. Monitoring sedation is essential in elderly patients for proper intraoperative management as over-sedation may jeopardise the safety of these patients [2]. For the assessment of sedation, a number of scales have been created to measure the patient's level of sedation as it progresses from consciousness to unconsciousness. The Ramsay sedation scale (RSS) is a well-established monitoring method to determine the level of awareness [3]. The RSS has flaws in patients with complicated conditions, despite its widespread usage. Many patients appear to adhere to more than one degree of sedation when the scale is administered at the bedside. For example, the patient may appear to be sleeping, with a slow reaction to the glabellar tap, yet be restless and worried at the same time. However, because of its simplicity, the scale can be used at remote locations.

The bispectral index (BIS) is a neurophysiological monitoring method using electroencephalograms for the continuous objective assessment of the depth of sedation [4]. There is some evidence from surgical patients that the bispectral index correlates well with the depth of sedation using propofol or midazolam [2]. Deep sedation can be avoided as evaluated by the BIS. It has been observed that BIS values differed at the same level of sedation between different sedative agents. We compared the correlation between the RSS and BIS in patients receiving either intravenous midazolam with patients receiving intravenous propofol for sedation in elderly patients under spinal anesthesia.

## Materials and methods

The study was conducted in a multispeciality corporate hospital after approval from the institutional ethical committee and review board. Informed written consent was taken from all the patients.

Patient selection

ASA grade I and II patients with the age group of >65 years undergoing urological surgeries under spinal anesthesia were included. Patients with a history of obstructive sleep apnea, drug addiction, difficulty in communication, and any contraindication to spinal anesthesia were excluded from the study.

Protocol

It was a prospective, single-centric, randomized study. The method of randomization was a computer-generated random number sequence that was sealed in opaque envelopes. The study included 60 patients who were randomly allocated into two groups. A detailed pre-anaesthetic evaluation was done, and informed consent was taken from all the patients. Injection pantoprazole 40 mg and ondansetron 4 mg were given to the patients in the pre-operative area. After confirming adequate fasting, the patient was taken inside the operation theatre (OT). Inside the OT, standard monitoring was attached, and intravenous access was secured. The BIS electrodes were placed on the forehead. Following baseline readings of vitals, the BIS and RSS were recorded (Table 1). Spinal anaesthesia was given in a sitting position with 26 G Quincke’s spinal needle using 0.5% hyperbaric bupivacaine (2.8 mL), along with an injection of fentanyl 20 mcg as an adjuvant. After checking adequate sensory block using an alcohol swab till the T10 level of dermatome distribution, positioning for surgery was allowed. Oxygen was administered using nasal prongs at 2-3 L/min.

In Group A, patients were given an initial bolus of midazolam 0.03 mg/kg and a maintenance incremental bolus of 0.01 mg/kg up to a maximum of 2.5 mg in a 10-minute interval. In Group B, propofol was given with an initial bolus dose of 0.5 mg/kg over two minutes and a maintenance bolus of 10-20 mg as required for the maintenance of sedation depth. Sedation was titrated to achieve a BIS score of 70-80, and the RSS score was noted at that time. Heart rate, non-invasive systolic, diastolic, mean arterial blood pressure, oxygen saturation (SpO2), BIS, and RSS were measured at baseline, 5, 10, 20, 30, 40, 50, and 60 minutes of intervals. After the procedure ended or at 50 minutes of duration, which was earlier, the study drug was stopped. The patient was shifted to recovery after the patient was alert and oriented, with a baseline saturation of more than 95% on room air and stable vitals.

**Table 1:** Ramsay sedation scale

Score	
1	Patient anxious, agitated, or restless
2	Patient cooperative, oriented, or tranquil
3	Patient responds to commands only
4	Brisk response to a light glabellar tap or loud auditory stimulus
5	Sluggish response to a light glabellar tap or loud auditory stimulus
6	No response

Statistical analysis

The statistical analysis was carried out using Statistical Product and Service Solutions (SPSS, version 25.0) (IBM SPSS Statistics for Windows, Armonk, NY). Measures of central tendency were used to measure all the quantitative variables. The normality of data was checked using appropriate tests of normality such as the Kolmogorov-Smirnov test. For normally distributed data, an unpaired t-test was applied. Data were expressed as mean and standard deviation for normally distributed continuous variables, or median (inter-quartile range) for non-normally distributed continuous variables. An independent t-test was applied to compare the mean of the normally distributed quantitative variables. For skewed data, the median was calculated, and the Mann-Whitney test and Wilcoxon tests were applied. The chi-square/Fisher's exact test was applied to find any significant association between the two study subgroups. Dunnett's multiple comparisons test was used to compare multiple means. P was considered significant at values where p<0.05.

## Results

The demographic characteristics such as age, sex, and weight were comparable between the two groups (Table 2). The duration of surgery and the type of surgeries undergone were also similar between the two groups (Table 3). Baseline vitals were comparable between the groups. The heart rate was significantly different at 5, 10, 20, 30, 40, and 60 minutes of the study (Table 4). The mean arterial pressure difference was significant at the five-minute and 40-minute duration (Table 5). The oxygen saturation remained optimum in both Group A and Group B from beginning to end, except at 20 minutes when the difference was significantly less in Group B (p=0.0225*). However, the oxygen saturation remains normal throughout the surgery.

**Table 2:** Demographic characteristics

Variable		Group A (Midazolam)	Group B (Propofol)	p value
Age (Mean ± SD)		71.90 ± 6.23 Years	71.37 ± 4.21 Years	0.6994
Sex (N, %)	Males	28 (93.33%)	24 (80%)	0.1287
	Females	2 (6.66%)	6 (20%)	
Body Weight (Mean ± SD)		76.80 ± 4.11 Kg	74.47 ± 6.25 kg	0.0930

**Table 3:** Duration and type of surgeries

Variable		Group A (Midazolam)	Group B (Propofol)	p value
Duration of Surgery (Mean ± SD)		54.00 ± 4.807 Minutes	51.50 ± 6.318 Minutes	0.0899
Type of Surgery (N, %)	Cystoscopy + TURP	12 (40%)	7 (24.13%)	0.5856
	Cystoscopy + TURBT	6 (20%)	4 (13.79%)	
	DIAG cystoscopy + TURP	5 (16.66%)	8 (27.58%)	
	DIAG cystoscopy + TURBT	1 (3.33%)	2 (6.66%)	
	TURBT + DJ stenting	1 (3.33%)	2 (6.66%)	
	TURBT + Prostate BX	0	1 (3.33%)	
	Urethral Dilatation + Cystoscopy	0	1 (3.33%)	
	URS	0	2	
	HOLEP	2 (6.66%)	1 (3.33%)	
	Cystoscopy + Clot Evacuation	1 (3.33%)	1 (3.33%)	
	Cystoscopy + TURP + TURBT	1 (3.33%)	0	
	Cystoscopy + TURP + OIU	1 (3.33%)	0	

**Table 4:** Heart rate at various time intervals of the study ***p value <0.001 (very strongly significant)

Duration (Minutes)	Heart Rate (Group A)	Heart Rate (Group B)	p value
Baseline	84.60 ± 7.445	81.70 ± 7.405	0.1358
5	74.77 ± 8.605	65.23 ± 4.804	0.0001***
10	71.67 ± 5.222	64.20 ± 4.318	0.0001***
20	70.83 ± 5.778	66.13 ± 4.439	0.0008***
30	72.03 ± 5.816	68.70 ± 4.276	0.0142*
40	73.47 ± 4.826	69.77 ± 7.190	0.0227*
50	74.27 ± 4.017	71.60 ± 7.180	0.0811
60	76.10 ± 3.188	73.60 ± 5.928	0.0465*

**Table 5:** Mean arterial pressure (MAP) at various time intervals of the study **p value <0.01 (strongly significant) *p value <0.05 (significant)

Duration (Minutes)	Mean Arterial Pressure (Group A)	Mean Arterial Pressure (Group B)	p value
Baseline	99.12 ± 5.094	96.57 ± 4.982	0.0543
5	87.04 ± 7.117	81.17 ± 9.106	0.0072**
10	84.00 ± 8.211	82.19 ± 6.823	0.3567
20	83.02 ± 8.166	82.94 ± 5.296	0.9652
30	89.11 ± 5.256	89.76 ± 5.784	0.6532
40	89.34 ± 5.648	85.68 ± 5.567	0.0141*
50	86.29 ± 4.391	84.41 ± 4.801	0.1193
60	89.88 ± 5.188	90.30 ± 3.894	0.7251

After giving the study drug as the initial loading dose in each group, we recorded the BIS value at five-minute intervals and then the RSS score at that time. Similarly, we followed these measurements for 60 minutes. The mean BIS score kept on decreasing, and the RSS score increased from the beginning to the end of surgery after maintaining the desired sedation in both Group A and Group B. Our range for the BIS was 70-80±5. BIS values were different between patients who responded to voice (RSS 3) and patients responding to light glabellar tap (RSS 4). The correlation analysis between the BIS and RSS scores in Group A at various time intervals indicate a strong correlation coefficient of -0.76 at five minutes (p=0.0001*), -0.64 at 20 minutes (p=0.0001*), -0.78 at 30 minutes (p=0.0001*), -0.56 at 40 minutes (p=0.0001*), and -0.39 at 50 minutes of interval (p=0.0014*) (Table 6). On the other hand, in Group B, the correlation analysis between the BIS and RSS scores at various time intervals indicates a strong correlation coefficient of -0.75 at five minutes (p=0.0001*), -0.76 at 20 minutes (p=0.0001*), -0.64 at 30 minutes (p=0.0001*), -0.89 at 40 minutes (p=0.0001*), and -0.46 at 50 minutes of interval (p=0.0095*) (Table 7).

**Table 6:** Correlation between bisprectral analysis (BIS) and Ramsay sedation scale (RSS) in Group A (midazolam) ***p value <0.001 (very strongly significant) **p value <0.01 (strongly significant)

Duration (Minutes)	Group A (Midazolam)			
	BIS Score	RSS Score	Correlation Coefficient	p value
0	98.90 ± 0.5477	1.867 ± 0.3457	-0.07	0.70
5	75.17 ± 3.896	3.400 ± 0.4983	-0.76	0.0001***
10	71.57 ± 3.451	3.767 ± 0.4302	-0.28	0.134
20	73.70 ± 4.112	3.367 ± 0.4901	-0.64	0.0001***
30	71.47 ± 3.902	3.667 ± 0.4795	-0.78	0.0001***
40	73.10 ± 4.596	3.345 ± 0.5526	-0.56	0.0001***
50	80.00 ± 3.494	3.345 ± 0.5526	-39	0.0014**
60	91.57 ± 3.501	2.000 ± 0.000	NA	NA

**Table 7:** Correlation between bispectral analysis (BIS) and Ramsay sedation scale (RSS) in Group B (propofol) ***p value <0.001 (very strongly significant) *p value <0.05 (significant)

Duration (Minutes)	Group B (Propofol)			
	BIS Score	RSS Score	Correlation Coefficient	p value
0	99.00 ± 0.000	1.800 ± 0.4068	NA	NA
5	73.17 ± 3.119	3.533 ± 0.5074	-0.75	0.0001***
10	68.33 ± 1.988	4.000 ± 0.000	NA	NA
20	71.33 ± 3.809	3.633 ± 0.4901	-0.76	0.0001***
30	73.17 ± 2.755	3.333 ± 0.4795	-0.64	0.0001***
40	71.80 ± 3.178	3.633 ± 0.4901	-0.89	0.0001***
50	77.27 ± 3.591	3.100 ± 0.3051	-0.46	0.0095*
60	93.37 ± 4.437	2.000 ± 0.000	NA	NA

## Discussion

Unfamiliar environments, anxiety, and loud noises in the perioperative period may have a long-term impact on the psychology of the patients [5]. In spinal anesthesia, the patient remains uneasy due to heaviness in the anaesthetised area, and the provision of good sedation improves the patient's comfort [6]. As more and more elderly patients are coming for surgeries due to increased life expectancy, the need for monitoring sedation becomes more important due to their decreased functional reserve. Over-sedation may jeopardize the safety of these patients. It makes accurate neurologic evaluation difficult and may cause cardiovascular instability. Hence, monitoring sedation in patients undergoing surgery is very important. The BIS helps in monitoring the depth of anaesthesia. Its value ranges from 100 to 0, with 100 denoting an awake clinical condition and 0 denoting absolute electric quiet (complete cortical suppression). General anaesthesia is defined as a range of 40-60, profound sedation is defined as a range of 60-70, and light to moderate sedation is defined as a range of 70-90. For readings more than 90, the patient is deemed awake [7]. For conscious sedation, a range of 65-85 has been proposed to decrease the danger of under or over-sedation. Another method that is extensively used in monitoring for determining the state of awareness is the RSS. It is checked by the patient’s response to a command to open their eyes or with a light glabellar tap. The problem with this scale is that it needs interaction with the patient and may awaken the patient. It categorizes a patient's sedation into six levels, ranging from extreme agitation to deep unconsciousness [8]. Despite its widespread usage, the RSS has flaws in patients with complicated conditions. Many patients appear to adhere to more than one degree of sedation when the scale is administered at the bedside. For example, the patient may appear to be sleeping, with a slow reaction to the glabellar tap, yet be restless and worried at the same time. However, because of its simplicity, the scale can be used at remote locations.

Under spinal anesthesia, hypotension occurs due to vasodilation as a result of loss of sympathetic tone. We found that MAP and HR were lower in patients receiving propofol than midazolam, and the difference was statistically significant. Propofol attenuates the baroreceptor reflex and blunts the reflex of tachycardia in response to a fall in blood pressure, leading to a lower HR in Group B patients. Meanwhile, doses of midazolam do not affect MAP and HR significantly. Similar hemodynamic responses were recorded in previous studies by Yaddanapudi et al. and Bagchi et al. [9,10]. However, Patki et al. observed that both propofol and midazolam in sedative infusions did not significantly alter mean heart rate or mean arterial blood pressure throughout the procedure [2]. The difference in their result as compared to ours may be due to the fact that the mean age of the patients in their study was younger and the hemodynamic response may differ from that of an elderly population. The oxygen saturation remained optimum in both groups from the beginning to the end of surgery in our study. The results of our study were in accordance with the results of the study by Patki et al., who also reported that neither propofol nor midazolam infusion, caused any significant alteration in mean respiratory rate or mean SpO2 throughout the procedure [2]. Both these drugs are known to depress respiratory function when given in inducing doses [2]. The cardio-respiratory function stability seen with both the drugs in our study can be possibly attributed to the fact that they were administered in sub-aesthetic doses.

The hemodynamics between the groups showed significant differences though the absolute values were always well within the normal limits. It showed that both propofol and can be used safely for sedation in elderly patients under spinal anesthesia.

Correlation between the BIS and RSS

Correlation is a measure to determine an association between different variables. If the data correlate, it means that the change in the magnitude of one variable is associated with a change in the magnitude of another variable, either in the same (positive correlation) or in the opposite (negative correlation) direction. A monotonic association is measured with Spearman rank correlation, which ranges from -1 to +1, where 0 indicates that there is no linear association or a constantly increasing or decreasing correlation as the coefficient approaches an absolute value of 1 [11-13]. We observed that the correlation between the BIS and RSS at five minutes was 76% in the midazolam group vs 75% in the propofol group. At 20 minutes, the correlation was 64% in the midazolam group vs 76% in the propofol group. The correlation at 40 minutes was 56% in the midazolam group versus 89% in the propofol group, whereas, at 50 minutes, the correlation was 39% in midazolam versus 46% in the propofol group. We concluded that the correlation between the BIS and RSS is more significant and stronger in Group B (propofol) as compared to Group A (midazolam) in our study.

Several studies have investigated the compliance between the BIS and clinical scales. Correlations have been determined at varying degrees in the published studies. Avci et al. [14] measured the accuracy of the BIS and RSS who had procedural sedation and analgesia in the emergency department. A high correlation was observed between the whole BIS and RSS scores at all measurements (r=-0.989, p<0.001). A moderate correlation was noted between the BIS and RSS scores of these 54 patients at the start of the procedure and five and 15 min after the procedure (r=-0.634, -0.637, and -0.665, respectively), and a high-degree correlation was observed at the start of the procedure and 10 and 20 min after the procedure (r=-0.748 and -0.774, respectively) [14]. In a study by Gill et al., a moderate correlation was observed between the BIS and modified RSS in 37 adult patients in the ED. In this study, the best BIS score that distinguished moderate sedation level from deeper sedation level was found to be 80 (sensitivity 86%, specificity 94%) [15]. Similar to the present study, Agrawal et al. [16] also studied a high-grade correlation between the BIS and modified RSS in 20 pediatric patients who underwent PSA. In the study by Yang et al., a weak correlation was detected between the RSS and BIS scores in 1,766 patients who underwent minor interventions out-of-surgery room by providing moderate sedation with midazolam [17]. Bell et al. compared the RSS with BIS assessment for the measurement of conscious sedation in interventional procedures. There was a significant correlation between the BIS and RSS [18]. A similar correlation between BIS and the Richmond agitation sedation scale has been done by Zheng et al. in the intensive care unit [19]. In our study, all the patients were above 65 years of age under spinal anesthesia, and we observed that the BIS and RSS are correlated more in the group receiving propofol for sedation rather than midazolam.

In elderly patients, as it is essential to monitor the depth of sedation to ensure safety, the RSS is a cost-effective tool when facilities for BIS monitoring are not available. It correlates well with BIS while using either propofol or midazolam for sedation. Our findings showed that BIS and RSS values are negatively correlated with an average magnitude of 70%-80%. However, the correlation is greater when using propofol for sedation as compared to midazolam. Thus, in all elderly patients under spinal anaesthesia, the RSS can be an effective alternative to monitor the depth of sedation comparable to BIS.

Limitations of the study

Despite our best possible efforts, there always remain some limitations that cannot be ignored. It was a single-center prospective study with a limited sample size. Therefore, the results could not be generalised until tested on a large stratum of the population.

## Conclusions

Monitoring the depth of anesthesia is very important in the elderly population to ensure safety and avoid over-sedation. We proposed that, in rural settings where there is a lack of instrumentation and expertise for the BIS score assessment, the RSS score could be used to map the level of sedation with an accuracy level of 70%-80% with a benchmarked BIS score. Therefore, the properties of each sedative drug should be considered when evaluating the level of sedation during spinal anaesthesia. Propofol and midazolam both are safe drugs when used for sedation in elderly patients with good hemodynamic stability and a good correlation between the RSS and BIS by using both the drugs. We propose that the RSS should be used to monitor depth in all elderly patients requiring sedation under spinal anaesthesia with either propofol or midazolam with accuracy similar to that of the BIS.
